# Stroke prediction in patients presenting with isolated dizziness in the emergency department

**DOI:** 10.1038/s41598-021-85725-1

**Published:** 2021-03-17

**Authors:** June-sung Kim, Hong Jun Bae, Muyeol Kim, Shin Ahn, Chang Hwan Sohn, Dong Woo Seo, Won Young Kim

**Affiliations:** Department of Emergency Medicine, University of Ulsan College of Medicine, Asan Medical Center, 88, Olympic-ro 43-gil, Songpa-gu, Seoul, 05505 Korea

**Keywords:** Neurology, Risk factors, Signs and symptoms

## Abstract

Diagnosing stroke in patients experiencing dizziness without neurological deficits is challenging for physicians. The aim of this study was to evaluate the prevalence of acute stroke in patients who presented with isolated dizziness without neurological deficits at the emergency department (ED), and determine the relevant stroke predictors in this population. This was an observational, retrospective record review of consecutive 2215 adult patients presenting with dizziness at the ED between August 2019 and February 2020. Multivariate analysis was performed to identify risk factors for acute stroke. 1239 patients were enrolled and analyzed. Acute stroke was identified in 55 of 1239 patients (4.5%); most cases (96.3%) presented as ischemic stroke with frequent involvement (29.1%) of the cerebellum. In the multivariate analysis, the history of cerebrovascular injury (odds ratio [OR] 3.08 [95% confidence interval {CI} 1.24 to 7.67]) and an age of > 65 years (OR 3.01 [95% CI 1.33 to 6.83]) were the independent risk factors for predicting acute stroke. The combination of these two risks showed a higher specificity (94.26%) than that of each factor alone. High-risk patients, such as those aged over 65 years or with a history of cerebrovascular injury, may require further neuroimaging workup in the ED to rule out stroke.

## Introduction

Patients experiencing dizziness commonly present at the emergency department (ED), accounting for roughly 5% of total visits^1^. Only a small portion of these ED cases are related to dangerous cardiovascular or cerebrovascular injury^2^. Acute dizziness is most often caused by benign otovestibular origins, including benign paroxysmal positional vertigo, vestibular neuritis, and Ménière's disease; however, it has also been implemented in the onset of stroke. Stroke is commonly misdiagnosed, noted as the fourth most common diagnostic error reported by physicians. Failure to recognize acute stroke may result in worse patient outcomes due to missed opportunity for acute stroke therapies^3^. Stroke diagnosis is particularly challenging in patients experiencing dizziness who have presented to the ED without neurologic deficits. There is evidence from previous research that roughly only 20% of patients diagnosed with acute stroke had focal neurologic abnormalities, and the frequency of misdiagnosis for stroke was up to 13%^4–6^. Clinical tools can help to reduce the risk of stroke misdiagnosis, examples of these include diffusion-weighted imaging (DWI) and magnetic resonance imaging (MRI) as the gold standard.

MRI can be useful in the exclusion of the central origin of dizziness in these patients; however, controversy exists on whether immediate requirement of MRI in the ED is necessary for patients presenting solely with dizziness because of the associated cost and limited access^7^. Although previous studies have reported some predictors and physical examinations (e.g. HINTS) for medical decision making, they were limited by their small sample size, performed MRI for patients with definite neurologic deficits, or did not include detailed medical records or conduct physical exams^8^. Given the large number of patients with dizziness presenting to the ED, further study to identify the predictors related to stroke will be needed in this population. The objective of this study was to evaluate the incidence of patients with dizziness without definite neurologic signs, and identify the relevant risk factors.

## Results

### Study population and baseline characteristics

**Figure 1:**
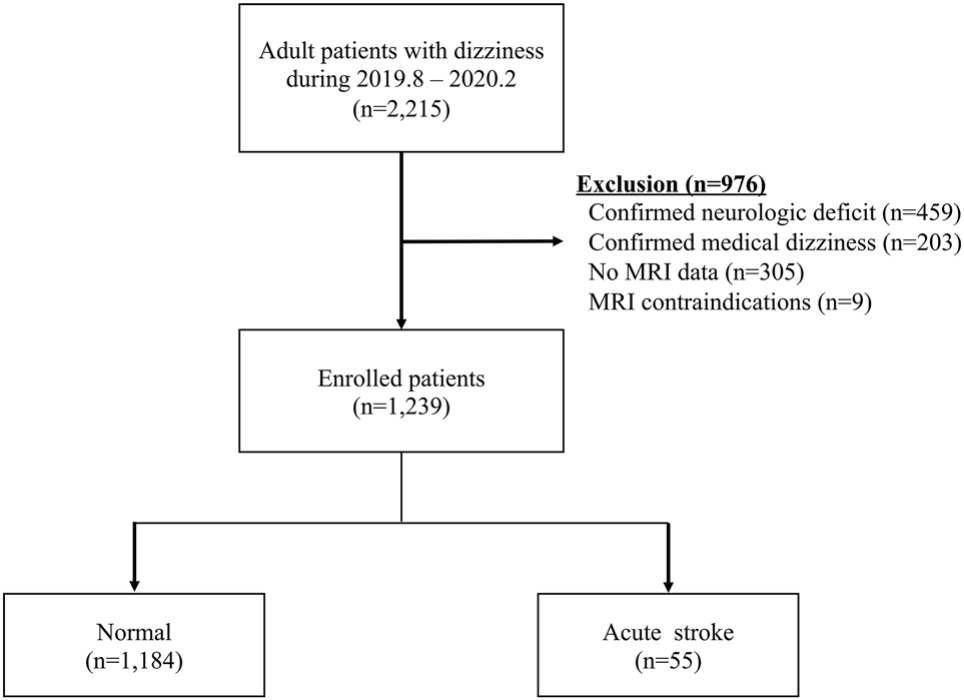
Flow chart of the study population. *MRI* magnetic resonance imaging.

During the study period, 2215 adult patients presenting with dizziness visited the ED. After excluding 456 patients with confirmed neurologic deficits, 203 patients were evaluated and diagnosed with confirmed clinical dizziness induced by, for example, anemia, hypoglycemia, shock, and electrolyte imbalance; 305 patients did not have MRI data, and 9 patients were contraindicated for MRI. The remaining 1239 cases were enrolled in the final analysis (Fig. 1). Of these, about 4.5% (55/1239) of patients were diagnosed with acute stroke. The median age of this population was 64.0 years (IQR 56.0–72.0) and 537 patients (43.3%) were male.

Baseline characteristics were compared, with patients categorized as either ‘patients with acute stroke’ or ‘normal’ (Table 1). Patients with acute stroke were older (64.0 vs. 71.5 years, *p* < 0.01), but sex preponderance was not identified. Patients with hypertension and atrial fibrillation were shown to have an increased risk of stroke compared with those without (56.4% vs. 42.2%, respectively, *p* = 0.04; 12.7% vs. 4.4%, respectively, *p* < 0.01), along with other comorbidities. Patients with a history of cerebrovascular injury were shown to be significantly associated with an increased risk of stroke compared with patients without (21.8% vs. 7.8%, *p* < 0.01). Medical use of anti-platelet or anti-coagulation agents had no significant impact on patient risk of stroke. With regard to characteristics of dizziness, dizziness with non-whirling quality (47.7 vs 63.6%, *p* = 0.02) and irrelevant to head movement (56.3 vs 72.7%, *p* = 0.02) was associated with acute stroke, but not other symptoms. Duration of symptoms were shown to not be significantly associated with the risk of acute stroke. Laboratory results (Table 2) showed that patients diagnosed with acute stroke had increased serum glucose levels compared with those who were not (136.5 vs. 123.0 mg/dL, respectively; *p* = 0.05). Creatinine levels between the two patient groups were similar (0.8 vs. 0.8 mg/dL, *p* = 0.01).

**Table 1:** Baseline characteristics of the study population.

Characteristics*	Total (n = 1239)	Normal (n = 1184)	Acute stroke (n = 55)	*p* value
Age	64.0 (56.0–72.0)	64.0 (56.0–72.0)	71.5 (62.0–76.8)	< 0.01
Male	537 (43.3)	507 (42.8)	30 (54.5)	0.09
**Past illness**				
Hypertension	531 (42.9)	500 (42.2)	31 (56.4)	0.04
Diabetes mellitus	198 (16.0)	188 (15.9)	10 (18.2)	0.65
Hyperlipidemia	265 (21.4)	248 (20.9)	17 (30.9)	0.08
Coronary artery disease	110 (8.8)	106 (8.9)	4 (7.3)	0.67
Atrial fibrillation	59 (4.8)	52 (4.4)	7 (12.7)	< 0.01
Chronic pulmonary disease	31 (2.5)	31 (2.6)	0 (0.0)	0.22
Malignancy	141 (11.4)	132 (11.1)	9 (16.4)	0.23
Cerebrovascular accident	104 (8.3)	92 (7.8)	12 (21.8)	< 0.01
Chronic kidney disorder	40 (3.2)	39 (3.2)	1 (1.8)	0.55
Liver cirrhosis	33 (2.7)	30 (2.6)	3 (5.4)	0.19
**Taken drugs**				
Aspirin	148 (12.0)	139 (11.8)	9 (16.4)	0.30
Clopidogrel	92 (7.4)	84 (7.1)	7 (12.7)	0.26
New oral anticoagulants	30 (2.4)	28 (2.4)	2 (3.6)	0.55
Warfarin	19 (1.5)	18 (1.5)	1 (1.8)	0.86
**Symptoms**				
Non-whirling	600 (48.4)	565 (47.7)	35 (63.6)	0.02
Wax and wane	325 (26.3)	316 (26.8)	9 (16.4)	0.40
Continuous	88 (7.1)	86 (7.3)	2 (3.6)	0.31
Irrespective of head movement	706 (57.0)	666 (56.3)	40 (72.7)	0.02
Headache	162 (13.1)	156 (13.2)	6 (10.9)	0.62
Nausea and vomiting	773 (62.4)	739 (62.5)	34 (61.8)	0.92
Tinnitus	130 (15.9)	128 (16.4)	2 (5.7)	0.09
Within seconds	20 (1.6)	18 (1.5)	2 (3.6)	0.22
Within minutes	120 (9.7)	115 (9.7)	5 (9.1)	0.88
Within hours	593 (47.9)	569 (48.1)	24 (43.6)	0.52
Within days	506 (40.8)	482 (40.7)	24 (43.6)	0.67

*Data are presented as n (%) or median with interquartile range.

**Table 2:** Laboratory results and EKG findings of the study population.

Characteristics	Total (n = 1239)	Normal (n = 1184)	Acute stroke (n = 55)	*p* value
WBC (× 10^3^/μL)	6.8 (5.5–8.5)	6.8 (6.6–8.4)	7.3 (5.4–9.2)	0.18
Hemoglobin (g/dL)	13.5 (12.5–14.5)	13.5 (12.0–14.5)	13.5 (12.2–14.6)	0.95
PLT (× 10^3^/μL)	225.0 (189.0–265.0)	226.0 (190.5–266.0)	211.5 (165.3–264.8)	0.37
Glucose (mg/dL)	123.0 (106.0–149.0)	123.0 (106.0–148.0)	136.5 (112.5–163.3)	0.05
BUN (mg/dL)	15.0 (12.0–19.0)	15.0 (12.0–18.0)	16.5 (13.3–19.0)	0.10
Creatinine (mg/dL)	0.8 (0.7–1.0)	0.8 (0.7–1.0)	0.8 (0.8–1.0)	0.01
Sodium (mmol/L)	140.0 (139.0–142.0)	141.0 (139.0–142.0)	140.0 (138.3–142.0)	0.17
Potassium (mmol/L)	4.2 (4.0–4.4)	4.2 (4.0–4.4)	4.1 (3.9–4.4)	0.58
Chloride (mmol/L)	103.0 (101.0–105.0)	103.0 (102.0–105.5)	103.0 (101.0–104.8)	0.31
LD (IU/L)	230.0 (203.0–263.0)	229.0 (203.0–262.0)	233.5 (202.0–275.5)	0.47

Data are presented as n (%) or mean with standard deviation.

*BUN* blood urea nitrogen; *EKG* electrocardiogram; *LD* lactate dehydrogenase; *PLT* platelet; *WBC* white blood cell.

The etiology based on Trial of ORG10172 in Acute Stroke Treatment (TOAST) classification, frequencies of vascular territories, and brain lesions are presented in Table 3. Of the total 55 patients, 52 (96.3%) experienced ischemic stroke. Large artery atherosclerosis was the most common subtype (40.8%), and vertebrobasilar artery (21.2%) was the most frequently involved culprit artery, followed by the middle cerebral artery (7.7%). The cerebellum was the most frequent brain lesion in acute stroke patients with isolated dizziness (29.1%), followed by pons (10.9%), and the frontal lobe (9.1%).

**Table 3:** Frequencies of vascular territories and brain lesions among patients with acute stroke on images.

Vascular territory	Numbers^a^ (%)	Diagnosis	Numbers^b^ (%)
Large artery atherosclerosis	21 (40.4)	Parietal lobe	4 (7.3)
Anterior cerebral artery	1 (1.9)	Occipital lobe	2 (3.6)
Middle cerebral artery	4 (7.7)	Frontal lobe	5 (9.1)
Posterior cerebral artery	2 (3.8)	Thalamus	1 (1.8)
Posterior inferior cerebellar artery	3 (5.8)	Cerebellum	16 (29.1)
Vertebrobasilar artery	11 (21.2)	Medulla	4 (7.3)
Multiple arteries	2 (3.8)	Pons	6 (10.9)
Small vessel disease	11 (21.2)	Hemorrhage	3 (5.5)
Embolic	12 (23.1)	Multiple	4 (7.3)
Undetermined	14 (26.9)	Other^c^	10 (18.2)

^a^Patients with ischemic stroke (n = 52) were included in the analysis.

^b^Patients with both ischemic and hemorrhagic stroke (n = 55) were included in the analysis.

^c^Included basal ganglia, corona radiata, internal capsule, midbrain, temporal lobe.

### Risk factors for acute stroke

The results of our univariable and multivariable logistic regression analyses to explore and identify risk factors for acute stroke in patients with dizziness are presented in Table 4. A history of hypertension (OR 1.77; CI 1.02 to 3.05, *p* = 0.04), atrial fibrillation (OR 3.18; CI 1.37 to 7.36, *p* < 0.01), cerebrovascular injury (OR 3.31; CI 1.69 to 6.50, *p* < 0.01), non-whirling type vertigo (OR 1.92; CI 1.09 to 3.36, *p* = 0.02), dizziness irrespective of head movement (OR 2.07; CI 1.13 to 3.79, *p* = 0.02), and age above 65 years (OR 2.68; CI 1.48 to 4.85, *p* < 0.01) were all factors associated with acute stroke in univariable logistic regression. A history of cerebrovascular injury (OR 3.08; CI 1.24 to 7.67, *p* = 0.02), and age above 65 years (OR 3.01; CI 1.33 to 6.83, *p* < 0.01) were the significant independent risk factors for predicting acute stroke in the multivariable logistic regression model.

**Table 4:** Univariable and multivariable analysis for predicting acute stroke.

Variables	Univariable			Multivariable		
	OR	95% CI	*p* value	Adjusted OR	95% CI	*p* value
Male	1.60	0.93–2.76	0.09			
HTN	1.77	1.02–3.05	0.04			
Hyperlipidemia	1.69	0.94–3.04	0.08			
CVA	3.31	1.69–6.50	< 0.01	3.08	1.24–7.67	0.02
Atrial fibrillation	3.18	1.37–7.36	< 0.01	2.40	0.82–7.01	0.11
Non-whirling	1.92	1.09–3.36	0.02	1.91	0.91–3.99	0.09
Irrespective of head positioning	2.07	1.13–3.79	0.02			
Tinnitus	0.31	0.07–1.31	0.09			
Age > 65	2.68	1.48–4.85	< 0.01	3.01	1.33–6.83	< 0.01
Glucose	1.39	0.77–2.51	0.27			
Creatinine > 1.5	2.31	0.78–6.78	0.12			

*CI* confidence interval; *CVA* cerebrovascular accidents; *HTN* hypertension; *OR* odds ratio.

The evaluation of diagnostic values of identified risk factors for acute stroke is presented in Table 5. Sensitivity, specificity, positive predictive value (PPV), negative predictive value (NPV), positive likelihood ratio (PLR) and negative likelihood ratio (NLR) of each risk factors were calculated. As predictive values for absence of acute stroke, history of without cerebrovascular injury showed high sensitivity of 78.18% but low specificity of 7.77%. Conversely, an age of under 65 years showed a sensitivity value of 36.36% and specificity value of 41.13%, which corresponds to a PPV and NPV of 2.79% and 93.30%, respectively. When the two risk factors were evaluated in combination, sensitivity performed better as a predictive factor (85.55%).

**Table 5:** Performance parameters for the absence of acute stroke in the study population.

Variables	Sensitivity (%)	Specificity (%)	PPV (%)	NPV (%)	PLR	NLR
No CVA	78.18	7.77	3.79	88.46	0.85	2.81
Age ≤ 65	36.36	41.13	2.79	93.30	0.62	1.55
No CVA + age ≤ 65	85.55	5.07	4.01	88.24	0.90	2.87

*CVA* cerebrovascular accident; *NLR* negative likelihood ratio, *NPV* negative predictive value; *PLR* positive likelihood ratio; *PPV* positive predictive value.

## Discussion

In this study, acute stroke was found in 55 of 1239 patients (4.5%) who presented with dizziness without confirmed neurologic deficits, and most cases (96.3%) were diagnosed as ischemic stroke with a high rate of cerebellum involvement. Although symptom characteristics and laboratory examinations could not be helpful to determine whether further neuroimaging workup was required, an age of over 65 (OR 3.01; 95% CI 1.33 to 6.83) and a previous history of cerebrovascular injury (OR 3.08; 95% CI 1.24 to 7.67) were independent risk factors for onset of acute stroke in this patient population.

Differentiating central from peripheral origins in dizziness is frequently challenging for physicians. In the past, isolated dizziness was considered as a peripheral origin disorder, meaning that patients experiencing this symptom had been excluded from most previous studies on acute stroke research^9^. On the contrary, there is a growing body of evidence to suggest that this isolated symptom is in fact caused by a central origin^10–12^. A retrospective study by Navi et al. reported that 37 of 907 (4.1%) patients who presented with dizziness also had cerebrovascular injury^12^. These data are similar with the data reported in this study (4.5%); however, Navi et al. included all patients with dizziness, irrespective of confirmed neurologic symptoms. Moreover, they found only two patients with isolated dizziness who were diagnosed with stroke and were limited by performing neuroimaging on only 37% of patients. A recent, small retrospective study reported that the incidence of stroke in admitted patients with isolated vertigo was about 11% (25/103) and was higher than in the present study. However, the smaller sample sizes of patients whom underwent MRI make it difficult to quantify the true incidence of stroke in these patients without neurologic deficits.

In this study, most cases of acute stroke were ischemic, and hemorrhage was relatively rare. Among 3 patients with hemorrhage, only 1 patient presented with dizziness without a headache, and the remainder suffered from a headache combined with moderately high systolic blood pressure (above 180 mmHg) at initial presentation. Large cohorts or systematic studies reporting on vertigo caused by hemorrhage do not appear to exist in the literature^13,14^, implying that patients with isolated dizziness were considerably less likely to have a primary intracranial hemorrhage. We also found that large artery atherosclerosis was the most frequent classification followed by small vessel occlusion and embolic origin according to the TOAST criteria. The incidence rates of each subtype were different compared with a past population-based epidemiological study^15^, which may be a result of the varying distribution of risk factors, including hypertension, diabetes, smoking rates, and a history of cardiac disease. However, because outcome data could be not collected, the differences in survival or recurrence rate according to each subtype could not be confirmed. Nevertheless, our findings confirmed the findings of the previous analysis that the cerebellum was the predominant site of ischemic stroke^16^. Additional brain sites affected included the frontal lobe, pons, and parietal lobe; these results added to the recent knowledge that dizziness as a symptom alone could not exclude the occurrence of stroke^17^.

We did not find any statistical differences between symptom characteristics and associated presentations. Although non-whirling type dizziness and irrelevance to head positioning appeared to be more prevalent in patients with stroke, these differences were not detected in the multivariate analysis. These results reflect those of the previous study that the effectiveness of symptom assessment in regard to patients with dizziness may be predisposed to under-evaluation of high-risk patients^18^. Numerous bedside oculomotor and neurologic examinations, such as Dix-Hallpike, head thrust, and HINTS +, would be a useful method for predicting a serious neurologic disease^19^. Alternatively, the results of these examinations tended to be reliable when patients were examined by trained neuro-ophthalmologists, and the consultations to the neurologists for all patients with dizziness may not be available in most clinical fields. Furthermore, we investigated the laboratory results that were usually performed to exclude medical dizziness (e.g., anemia, hypoglycemia, or electrolyte imbalance), and found that none of the data could discriminate the presence of stroke.

The correlation between older patient age and increased risk of stroke, including in patients without confirmed neurologic symptoms, was as predicted. Chase et al. also noted similar findings in their prospective study of patients presenting to the ED^20^. Nevertheless, other well-known risk factors, including diabetes mellitus, hyperlipidemia, and protective agents, such as aspirin, warfarin, and new oral anticoagulants, showed an association with the development of stroke in this study. Although atrial fibrillation did not appear to independent risk factor of stroke, we did find a trend toward significant association (adjusted OR 2.40, 95% CI 0.82 to 7.01). Traditionally, atrial fibrillation itself increased the risk of stroke events and recurrence by up to 54.0% in a previous study^21^. In addition, we found that the patients with acute stroke had higher proportion of previous cerebrovascular injury. Interestingly, our study population only included patients who had no long-term neurologic deficit after initial stroke presentation, but the recurrence risk increased compared with in patients without previous history of cerebral infarct. Combination of older age under 65 and without history of stroke had high sensitivity, but low specificity for predicting absence of acute stroke. Hence, patients with dizziness who are aged over 65 years and have a history of cerebrovascular injury should be considered for further neuroimaging tests.

We note several limitations of this study. Firstly, the single-center, retrospective design, has meant that we could not generalize our results to the wider environments. Secondly, patients who did not visit the ED or had no MRI data could have led to selection bias. Thirdly, relatively small sample of the patients with acute strokes might affect sensitivity analysis. In addition, there was no standardized protocol for the making decision of patients with dizziness. Therefore, misdiagnoses or delayed presentation of cerebrovascular injury may not have been detected in this analysis. Lastly, unmeasured confounding variables, such as smoking history, could have influenced the results.

## Conclusion

The incidence of acute stroke was 4.5% (n = 55/1239) in patients presenting to the ED with dizziness but without neurological deficits. Given the large number of these patients, it would be necessary to consider further neuroimaging workup in high-risk patients, such as those aged over 65 or those with a history of stroke, in order to rule out acute stroke as the cause.

## Materials and methods

### Study design and setting

This was a single-center, retrospective, observational study conducted in the ED of Asan Medical Center, an urban tertiary referral hospital in Seoul, Korea. Adult (> 18 years) patients who visited the ED with dizziness or related symptoms (e.g., vertigo, lightheadedness, disequilibrium, or gait instability) as the chief affliction between 1 August 2019 and 29 February 2020 were evaluated. The ED of the study facility is staffed 24 h a day by attending emergency medicine physicians and residents. Neurology consultants and neuroimaging facilities (brain computed tomography, MRI) are accessible around-the-clock. Patients who had triage complaints of dizziness were routinely evaluated with systemic history documentation, physical and neurologic examinations, and further laboratory tests; the necessity of follow-up neuroimaging examinations was determined by the primary emergency medicine physician on duty. All experiments were carried out in agreement with relevant described guidelines and regulations. This study was approved by the Research Ethics Committee of Asan Medical Center (no. 2020-0179) which waived the requirement for patient informed consent.

### Study population and variables

Electronic medical records were reviewed by trained researchers (H.J.B., and J.S.K.) and all eligible adult patients with isolated dizziness or vertigo were included in the study. Exclusion criteria included patients with confirmed neurologic deficits at presentation, including hemiparesis, hemiplegia, dysarthria, facial droop, and a Glasgow Coma Scale score of less than 13; patients with dizziness as a result of external clinical factors (e.g., hypoglycemia, shock, symptomatic bradycardia, sepsis, or intoxication); and those without MRI data.

In all confirmed cases of acute ischemic stroke, magnetic resonance angiography was performed for evaluation of vascular abnormality. MRI scans were interpreted by qualified radiologists, and associations between the ischemic territory and culprit artery were also investigated. Old ischemic lesions were not considered to be acute central lesions relevant to the current case of stroke. The etiology of acute stroke was evaluated by experienced neurologists on duty by use of TOAST criteria^22^. Notes on vascular territory and TOAST criteria were also extracted from medical records.

DWI (5 mm slice thickness, an interslice gap of 2 mm, 20 axial slices, and a field of view of 250 mm) was performed on a 1.5 T MRI unit with echoplanar capability (Signa CV/i; GE Medical Systems, Milwaukee, Wisconsin, USA). DWI parameters included a repetition time of 7500 ms, an echo time of 84 ms, a matrix number of 128 × 128, and a b value of 1000 s/mm^2^.

Data collected for the patients in our study included demographics, underlying medical illnesses, any current use of anti-platelet or anti-coagulation agents, characteristics of dizziness and associated symptoms including headache, nausea, vomiting, and tinnitus. Because of the retrospective design, we did not have a standardized case report form. However, the electronic medical records included information about demographics, underlying medical illnesses, and routine medications. Moreover, experienced primary physicians on duty or neurologists were well-trained in routinely recording characteristics of dizziness and associated symptoms for patients with dizziness in medical charts. Laboratory data were also collected for analysis, including white blood cell counts, hemoglobin, glucose, blood urea nitrogen, creatinine, and electrolyte profile.

### Statistical analysis

The continuous variables in the baseline demographics, clinical characteristics, and laboratory data were presented as medians with interquartile range (IQR) and as frequencies with percentages. The Kolmogorov–Smirnov test was used to validate the normality of distribution, and the Mann–Whitney U test was performed. Categorical variables were assessed using the chi-squared test or Fisher’s exact test, as appropriate. A stepwise multivariate logistic regression analysis was employed to identify risk factors that are predictive of acute stroke in patients experiencing dizziness. Variables with *p* values of less than 0.1 in univariable analyses were selected for the multivariable analysis. Cut-off values for continuous variables, including age and serum creatinine levels, were determined by use of the Youden index (sensitivity + specificity − 1), with a receiver operating characteristic curve. Sensitivity and specificity were calculated by use of the standard statistical method. *p* values lower than 0.05 were considered to indicate statistical significance in this study. All statistical analyses were performed using SPSS Statistics for Windows, version 26.0 (IBM Corp., Armonk, NY).
